# The method of radiation risk assessment based on physico-geographical regionalisation: a case study of Carpathians, Poland

**DOI:** 10.1007/s11356-024-35518-6

**Published:** 2024-11-25

**Authors:** Filip Jędrzejek, Katarzyna Szarłowicz, Marcin Stobiński

**Affiliations:** Faculty of Energy and Fuels, AGH University of Krakow, Al. Mickiewicza 30, 30-059 Kraków, Poland

**Keywords:** Gamma-ray spectrometry, NORM, Adsorbed dose rate, Annual effective dose, Health risk assessment, Terrestrial radiation

## Abstract

Radiological assessment of the environment in expansive territories poses significant challenges due to the complexity of spatial and environmental variables. The aim of this study was to use and present the advantages of a physico-geographical regionalisation methodology to improve the precision and effectiveness of radiological assessments in large areas. The study area was a region of the Polish Carpathian mountains with a territory of 19,600 km^2^. By integrating geographic information systems (GIS) and radiological data, this research analysed a similarity of environmental attributes (soil type, lithology, mineralogy) and absorbed dose rate (ADR) level from the terrestrial origin for populations. Consistency in terms of the nature and origin of the relief and lithological diversity was a key aspect that distinguished the radiological characteristics. Therefore, consistency was observed at the macroregional level according to physiographic mapping. The study demonstrates the utility of macroregionalisation in capturing spatial ADR heterogeneity and provides a new strategy in radiological monitoring.

**Graphical Abstract:**

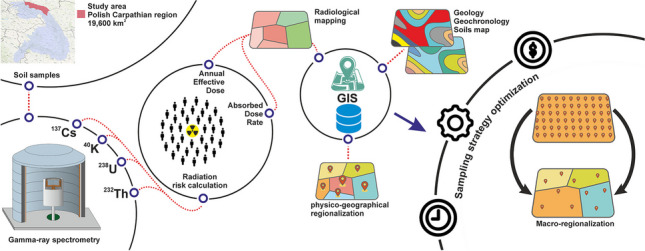

## Introduction

Ionising radiation is an essential element of natural environments and daily life. This is concatenated by the presence of radioactive elements present in nature. Due to the need for radiological protection of the population, exposure resulting from the presence of several radionuclides in the environment is monitored. Studies on radiation level and radionuclide distribution in the environment provide vital radiological baseline information on human exposure to natural and man-made sources of radiation that may be relevant to radiation protection (Arriola-Velásquez et al. 2024; Car et al. 2024; ICRP 2007; NRC 2006).

Natural environmental radioactivity depends on many factors, especially geological and geomorphological conditions. The distribution of radionuclides in an ecosystem is influenced by the chemical properties and physical factors of an ecosystem, as well as processes that take place in the area. That is why radionuclides appear at different levels in rocks, soils, or sediments in each region of the world (Ram et al. 2021). The external component of natural radioactivity can be divided into terrestrial and cosmic. Terrestrial radionuclides occur naturally in all parts of the environment. Among them are the radioactive families of uranium and thorium radioisotopes and their progeny. As well as primordial radioisotope of potassium ^40^ K (Blanco Rodríguez et al. 2008; Seaman and Roberts 2012). The bedrock is the main reservoir of the natural uranium radionuclides (especially ^238^U, ^235^U) and thorium (e.g. ^232^Th, ^228^Th) which are released into the soil through weathering processes (Cicchella et al. 2014). The mobility of uranium during weathering processes is highly dependent on host minerals (Kabata-Pendias 2010). Most thorium is concentrated mainly in resistive minerals such as monazite and zircon, so it tends to remain in soil profiles. When thorium is removed from the soil, it is transported to solid mineral compositions, as thorium is largely insoluble in surface and groundwater (Hem 1992). Potassium is the main component of the soil and is part of the mineral composition. Consequently, its concentration is essentially related to the bedrock. In most environmental conditions, K^+^ released from minerals by weathering is easily incorporated into structures, adsorbed by clay minerals, and/or readily absorbed by plants (Reimann 2000b; Saaltink et al. 2014).

In addition to naturally occurring radioactive materials, some human activities can also enhance the level of natural radiation background. This phenomenon is known as anthropogenic radioactivity, including artificial-type radionuclides and TENORM (Technologically Enhanced Naturally Occurring Radioactive Materials). Among artificial radionuclides, those from nuclear weapons tests (1950/1960) and nuclear accidents (e.g. Chernobyl, Fukushima) could be recognised. Each nuclear disaster causes the distribution of radionuclides, in an uncontrolled manner, and all parts (e.g. soils, water, sediments, air) of the environment are contaminated. The main source that changed the level of the Earth’s radiation background is the global fallout of long-lived radionuclides (e.g. ^137^Cs, ^90^Sr, ^238,239^Pu) resulting from nuclear testing or accidents (Anderson et al. 2022; Beresford et al. 2016; Hatamura 2015; Nakanishi et al. 2023). The major industrial sectors that generate TENORM are mining, energy production (coal combustion), consumer products (fertiliser), and water treatment (wastewater treatment residuals). During this, naturally occurring radionuclides are concentrated and redistributed into the environment (Bou-Rabee et al. 2009; Landa 2007; Luca et al. 2009; Szarlowicz et al. 2019; Vaasma et al. 2017).

The global average exposure of humans to ionising radiation is about 3 mSv per year, 80% of which comes from nature. The remaining 20% results from exposure to human-made radiation sources, primarily from medical imaging. Therefore, the highest contributor to the dose received by the world population comes from natural sources, and the assessment of background gamma radiation dose is of particular significance. In all of the world, routine environmental monitoring is carried out. Such monitoring involved the evaluation of the dose obtained by using radioactivity concentration in environmental media and measurements of external dose rates. It is designed specifically for each facility taking into account factors such as climate, site location, geological, and geomorphological conditions. Various ecosystems of the natural environment are taken into account; most often, radioactivity is measured in soils, water, and air. The sampling of soils, sediments, or deposits serves as an indicator of long-term build-up of radioactivity in the environment (Chernyaev et al. 2004; Eleftheriou et al. 2013; Kubica et al. 2014; Nowak and Solecki 2015; Szarlowicz et al. 2011; Videvall et al. 2023).

However, studies involving radiological assessment of large geographical areas could be a challenging project. Especially since this often involves regular monitoring, which requires many soil samples. The selection of survey points is usually based on a wide grid, where monitoring points are agglomerated within regions of a political subdivision, e.g. provinces. This approach is the most accurate for representing radiological conditions in various geopolitical areas. However, it requires a large amount of work to collect, prepare, and analyse representative samples. Therefore, this work proposes a different strategy to implement this type of research, based on macroregional division.

The macroregional subdivision is the result of interdisciplinary work related to physiographic cartography. Physiography refers to the study and description of the physical features and natural landscapes of the Earth’s surface, including its landforms, topography, geology, and hydrology. It encompasses the analysis of various factors that shape the Earth’s surface, such as tectonic activity, erosion, deposition, and weathering processes. Physiographic studies aim to understand the spatial distribution and arrangement of landforms and geological features across different regions. By examining the physical characteristics of landscapes, physiographers seek to decipher the underlying geological processes and historical events that have shaped local conditions. Their work integrates principles from various scientific fields, including geology, geomorphology, hydrology, climatology, and ecology (Butler and Marston 2017). The result of the work is a physical-geographical regionalisation, considering the division into provinces, macroregions, mesoregions, and micro-regions. Macroregions typically encompass extensive territories and include multiple intermediate-scale geographical areas, mesoregions (Frye et al. 2023).

The aim of this study was the assessment of ionising radiation health risk based on radionuclide determination in soil samples and to provide a new optimised strategy for selecting measurement points based on physiographical regionalisation. The objectives of the study are as follows: determine and evaluate the levels and distribution of gamma radionuclides in soil samples, calculate the annual effective dose, use ArcGIS software for preparing radiation maps, identify the key factors contributing to the annual effective dose interpret concentrations in macroregions based on mesoregions, and demonstrate the effectiveness of the application of macroregional division in the assessment of annual effective dose.

## Study area

The Carpathian mountains belong to one of the largest mountain ranges in Europe and run through the territories of eight countries. The area of the entire Carpathians Mountains is up to 209,000 km^2^ (CCIBIS 2022). It is characterised by a medium altitude, where the lowest point is 501 m above sea level (Dukla Pass), while the highest peak is 2655 m in altitude (Gerlach Peak). The geological structure of the Carpathians is very diverse and dominated by Carpathian flysch (sandstones and shales); less frequently, there are limestones and dolomites, extrusive rocks, granites, and metamorphosed rocks. The climate of the Carpathians is typical of mountainous areas, with an altitudinal zonation (Adamus 1995; Zemanek 2009). The research area was approximately 19,600 km^2^ (9.6% of the entire Carpathians) and covered the entire Polish Carpathian region.

## Materials and methods

**Fig. 1:**
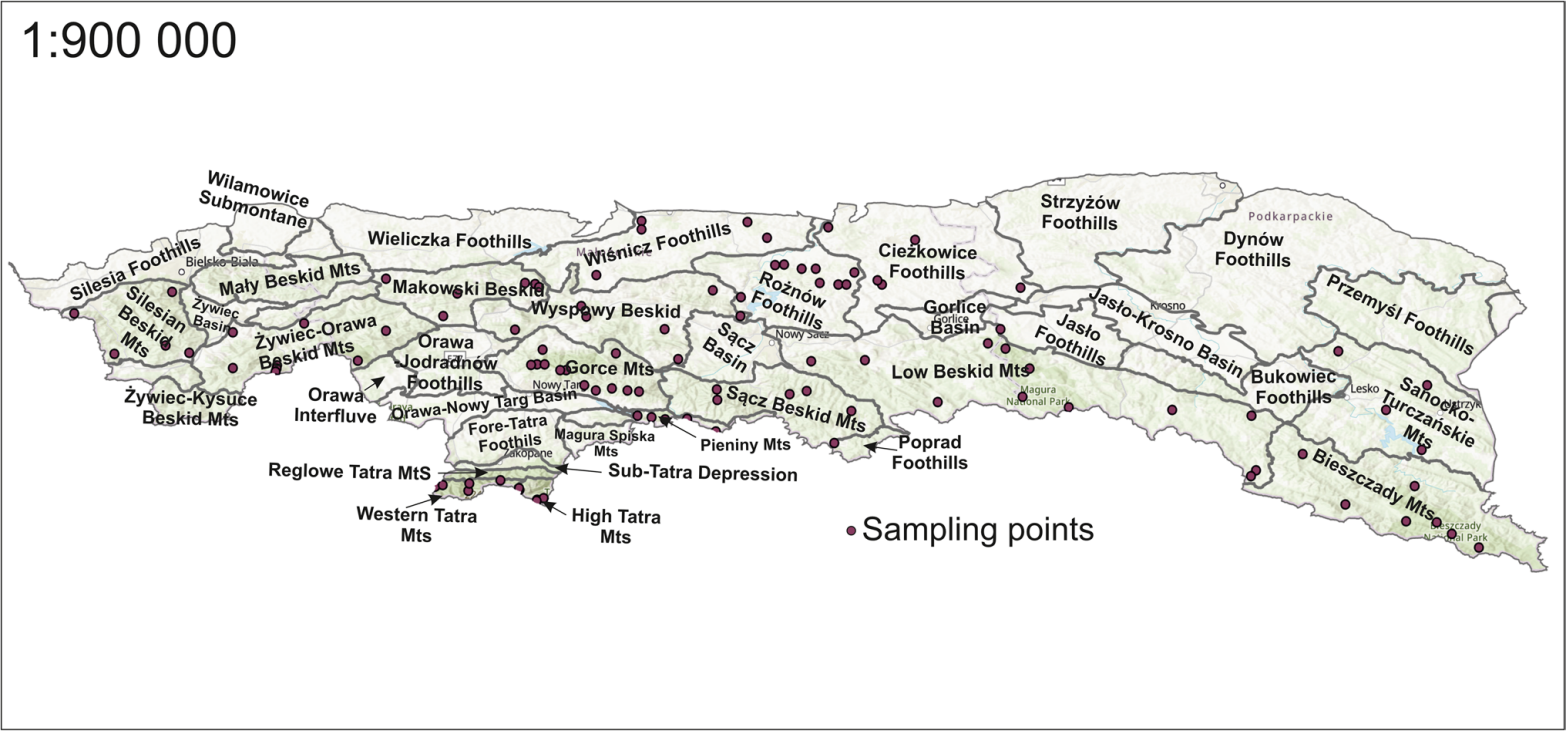
Soil sampling points in the Polish Carpathian area

The selection of sampling sites was made based on mesoregional divisions of the study area, using a strategy involving geological point diversification (Jędrzejek et al. 2022; Solon et al. 2018). In the mesoregions, points were selected where both the maximum and minimum levels of natural isotope radioactivity were predicted to occur. It was assumed that various types of rock specifically impact the concentration of radioactive isotopes, e.g. granite, granitoid, and gneiss or quartz sandstone. The extrema identified within the mesoregions were used to characterise the average value. Based on the distribution of these regions, more than 100 measurement points were selected and more than 300 soil samples were taken. All points are summarised and shown in Fig. 1.

Soil samples were taken to a depth of 10 cm, as proposed by the National Atomic Energy Agency (Agency 2017). Sampling was performed using cylindrical samplers with a diameter and length of 10 cm. GPS coordinates and altitude were recorded during the sampling. Then, 10-cm soil cores were placed in polyethylene bags and transported to the laboratory. In the laboratory, each core was divided into three layers, a, b, and c. Layer ‘a’ is the surface, layer ‘b’ is the middle layer, and layer ‘c’ is the deepest layer. After sampling, the soil was dried at 50 °C in the laboratory dryer. A few days later, the samples were weighed, homogenised, and sieved (diameter of the sieve 1 mm). The material obtained was transferred to a cylindrical measuring container made of transparent polypropylene, which had a volume of 27 cm^3^. For each vessel, the mass of the sample inside and its density were determined (knowing that the volume of the sample is exactly 27 cm^3^). The vessels were then sealed with parafilm to prevent exhalation of radon gas. After about a month, an equilibrium could be created between ^226^Ra, ^222^Rn, and other short-lived decay products (such as ^214^Pb and ^214^Bi) necessary to determine the radioactivity of ^226^Ra. After equilibrium was reached, the samples were measured on a gamma spectrometer equipped with an HPGe detector (high-purity germanium) with vertical dipstick cryostats. Two detectors (type-broad energy, BE3830) with a relative efficiency of 34% manufactured by Canberra (Mirion) were used. Each detector had shielding made of lead with an inner 10-cm layer of low background lead and graded cadmium and copper shield. One of them was also equipped with a special background reduction system (Jędrzejek and Szarłowicz 2024).

The reference materials used (produced by the International Atomic Energy Agency) were as follows: IAEA-447 (for artificial ^137^Cs, ^241^Am and natural ^40^ K), IAEA-RGTh-1 (for radioisotopes from the ^232^Th-series), and IAEA-RGU-1 (for radioisotopes from the ^238^U-series and for ^235^U). The determination of gamma radioisotopes ^137^Cs, ^241^Am, ^40^ K, ^228^Th, ^228^Ra, ^226^Ra, ^210^Pb, and ^238^U is based on the peak area of characteristic spectral lines and is described in detail in the literature (Stobiński et al. 2018).

The radioactivity of ^235^U cannot be determined directly because ^226^Ra has a spectral line with the same energy (186 keV), and in the spectrum, it is the sum of two spectral lines. Therefore, a specific procedure was used to determine this isotope (^235^U) (Ebaid 2010).

All measurements of ^137^Cs radioactivity due to its relatively short half-life were calculated on January 1, 2023. The measurement results take into account the self-absorption coefficients determined according to Misiak et al. (2011). The indicated measurement uncertainties are the result of the uncertainty of the reference materials, the uncertainty of the background and sample measurements, and the uncertainty of determining the detection efficiency.

In this work, the gamma radioactivity of ^137^Cs is also presented as radioactive concentration per square metre (expressed in Bq⋅m^−2^). The following formula was used for the conversion:

1 $${A}_{\text{s}} [\text{Bq}\cdot {\text{m}}^{-2}]=\frac{{A}_{\text{a}}[\text{Bq}\cdot {\text{kg}}^{-1}]\cdot {m}_{\text{a}}[\text{kg}]}{\text{0,0085}[{\text{m}}^{2}]}+\frac{{A}_{\text{b}}[\text{Bq}\cdot {\text{kg}}^{-1}]\cdot {m}_{\text{b}}[\text{kg}]}{\text{0,0085}[{\text{m}}^{2}]}+\frac{{A}_{\text{c}}[\text{Bq}\cdot {\text{kg}}^{-1}]\cdot {m}_{\text{c}}[\text{kg}]}{\text{0,0085}[{\text{m}}^{2}]}$$

where:

*A*_s_—radioactivity concentration of the isotope in the soil in Bq⋅m^−2^

*A*_a_, *A*_b_, *A*_c_—radioactivities of the isotope in particular layers of the soil

*m*_a_, *m*_b_, *m*_c_—total masses of the samples obtained after drying (described above) in particular layers

0.0085 m^2^—surface of the soil taken by the probe.

### AED calculation

To compare exposure to ionising radiation from radionuclides according to the UNSCEAR report, the AED (annual effective dose) parameter was calculated (UNSCEAR 2008). AEDs are indicators of direct exposure to external radiation generated by gamma and beta radiation related to the presence of NORM (naturally occurring terrestrial radionuclides) in the surface soil. NORMs occur in soils and rocks in various chemical forms. This includes the impact of the most significant radioisotopes of the Earth’s crust on the population’s exposure to ionising radiation.

The radioactivity concentration of the artificial ^137^Cs isotope, although it can vary within a wide range in soils, may be negligible in the AED calculations in relation to the radioactivity of all natural terrestrial radionuclides.

The AED is calculated based on the following formula (UNSCEAR 2008):

2 $$\text{AED }[\text{Sv}\cdot {\text{y}}^{-1}] =\text{ ADR }[\text{Gy}\cdot {\text{h}}^{-1}] 8760 [\text{h}]\cdot 0.2\cdot 0.7 [\text{Sv}\cdot {\text{Gy}}^{-1}]$$

where:

Value 8760 (h) is the number of hours in year

0.2 is the statistical factor of time spent in the open air

0.7 (Sv·Gy^−1^) is a factor to convert the effective dose rate into the absorbed dose rate in the air for adults

ADR (Gy·h^−1^) is the absorbed dose rate calculated based on the formula:

3 $$\text{ADR }= 0.462\text{AU }+ 0.604\text{ATh }+ 0.0417\text{AK}$$

where:

0.462, 0.604, and 0.0417 (nGy·h^−1^ per Bq·kg^−1^) are dose coefficients that relate soil concentrations to the absorbed dose rate in the air.

AU, ATh, and AK are the radioactivity concentration of radionuclide (Bq·kg^−1^) of ^238^U, ^232^Th_series_, and ^40^ K respectively.

### Geological information system

The maps were created in ESRI’s ArcGIS Desktop software package, using ArcMap 10.7.1 and ArcGIS Pro 3.2. The basemap layer was a topographic world map provided by ESRI, and for the study area, coverage was provided down to ~ 1:4 k (ESRI 2017).

A geographical coordinate system was used: GCS_European_1987.

The following data were used to prepare GIS analyses and map drawings:

1. Mesoregions and Macroregions of Poland (Solon et al. 2018)
2. Geological Map of Poland (PGI PGINRI 2022)
3. Generalized Geology of Europe (Pawlewicz et al. 2003)
4. Map of soils of Poland (Dobrzański et al. 1984)

## Results and discussion

### General radiological characteristics of the Carpathian mesoregions

This chapter presents mappings of the concentration distribution of selected radionuclides, which are presented as averages for the designated mesoregions. The distribution of concentrations of natural radioisotopes ^40^K, ^238^U, ^232^Th, and anthropogenic ^137^Cs is presented.

**Fig. 2:**
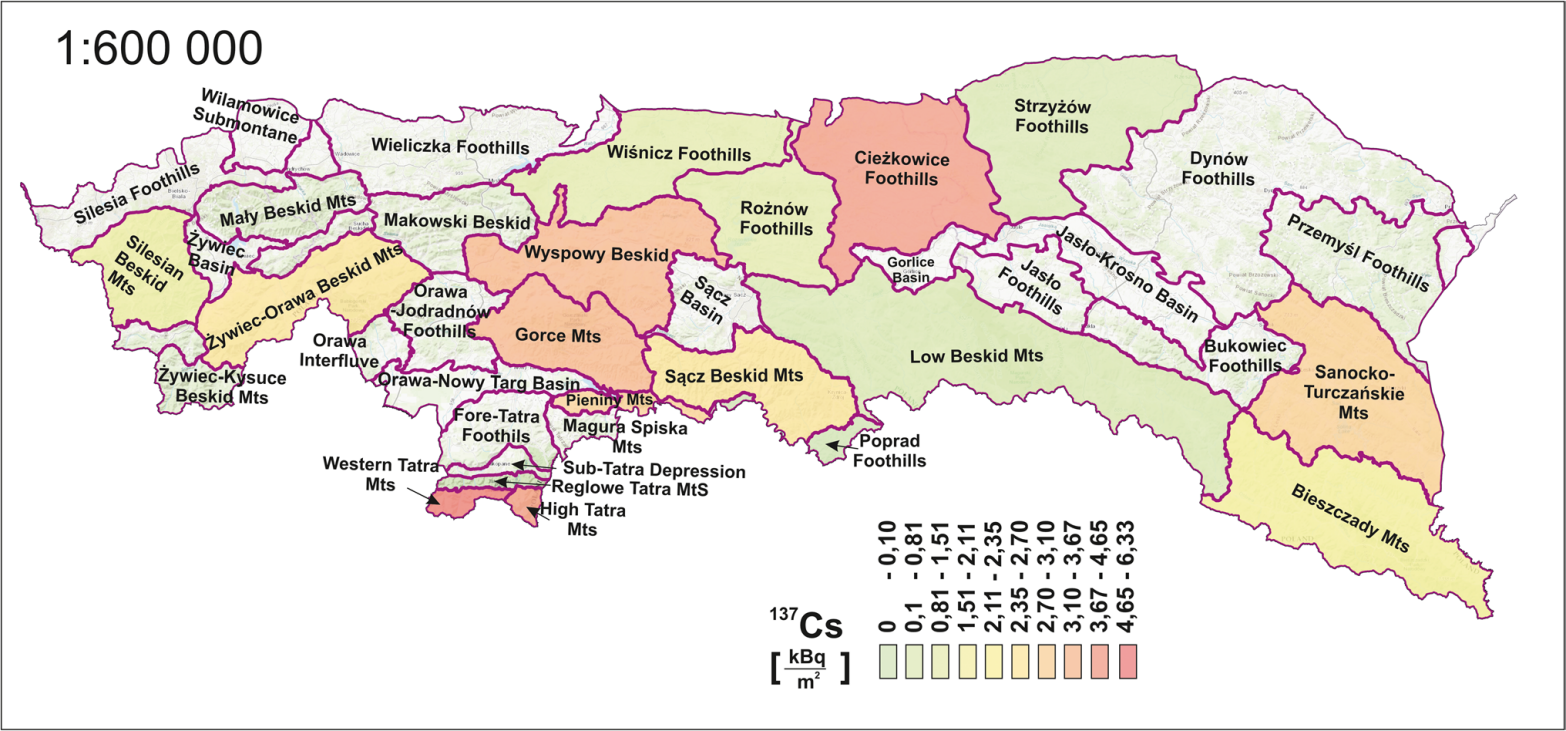
Spatial distribution of ^137^Cs radioisotope concentrations by mesoregion

Figure 2 shows the distribution of ^137^Cs concentrations. It was in the range of 0.1–6.33 kBq·m^−2^, with an average of 2.68 kBq·m^−2^ for the entire Polish Carpathian megaregion. These values are in the range of radioactivity calculated in soil samples for ionising monitoring (PAA PNAEA 2023). The data obtained are lower than those for the Romanian Carpathians (covering more than half of the area of the Carpathians), especially three times lower for mountain areas (Begy et al. 2017). The distribution of this radionuclide is highly variable due to deposition from the atmosphere, which is characterised by an extremely random nature. The distribution presented makes it impossible to apply any regionalisation. Therefore, a better strategy than a wide measurement grid is not applicable. However, a comment should be made here on the relevance of environmental concentrations of this radionuclide to the annual effective dose (AED), which is used to estimate population exposure. The AED was in the range of 0.0001–0.0079 mSv·y^−1^. The calculations have been made according to the guidelines of the UNSCEAR 2000 report, which provides the conversion from surface caesium concentration to effective dose. It resulted in a maximum share of 3% in total AED. The coefficients for natural sources are on the order of magnitude higher due to the addition of all radiogenic isotopes formed in series. Obviously, because of several studies related to the transport mechanisms of this radioisotope, thematic research is important. However, for population exposure, the impact of this radionuclide is negligible. Protection functions in this aspect are the stations of the early detection system for radioactive contamination in the air.

**Fig. 3:**
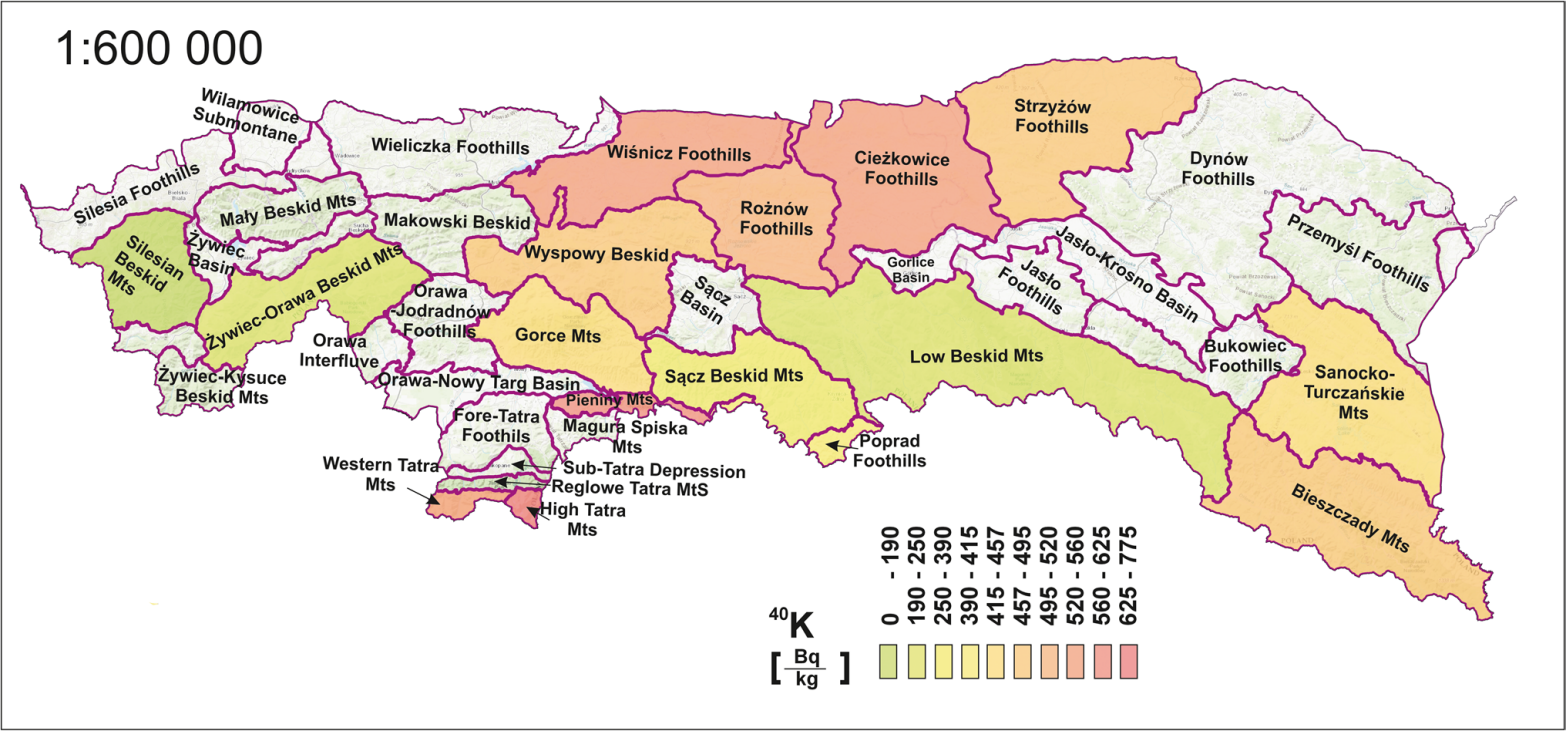
Spatial distribution of ^40 ^K radioisotope concentrations by mesoregion

Figure 3 shows the ^40^K concentration distribution and appears to be more uniformly dispersed than the caesium presented in the earlier figure. The average value calculated for the megaregion was 463 Bq·kg^−1^, and the values for individual mesoregions were in the range of 190–775 Bq·kg^−1^. The radioactivity of ^40^ K was of similar magnitude, when compared to studies in other parts of the Carpathians. In the Slovak part of the Carpathians (Žiar nad Hronom, Western Carpathians), ^40^ K radioactivity in soils was in the range of 31–1346 Bq·kg^−1^, with an average of 476 Bq·kg^−1^ (Porubčanová et al. 2015). The Romanian Carpathians were respectively 142–1163 Bq·kg^−1^, with an average 665 Bq·kg^−1^ (Blebea-Apostu et al. 2024). However, in the Ukrainian Carpathians indicated in the publication (Maslyuk et al. 2016), the average concentration was approximately 339 Bq·kg^−1^, and in the Czarnohora Mountains—411 Bq·kg^−1^ (Skiba et al. 2005).

Analysing the ^40^ K isotope distribution, it is possible to distinguish that the regions best representing the mean value adopted for the megaregion (i.e. oscillating up to a maximum of half a standard deviation around the mean) were in the eastern and north-eastern parts of the Carpathians. The central part of the range was the area for which the values are significantly below the average, these regions are Low Beskid Mts., Sącz Beskid Mts., Gorce Beskid Mts., Żywice-Orawa Beskid Mts., and Silesian Beskid Mts. In this Beskid range, potassium radioactivity gradually decreases from east to west, where a minimum value of 190 Bq·kg^−1^ was observed in the west (Silesian Beskid Mts). In the second south-north axis, a relationship could also be observed. The highest radioactivity of 775 Bq·kg^−1^ was obtained for the High Tatras region, 625 Bq·kg^−1^ for the Pieniny and 563 Bq·kg^−1^ for the Wiśnicz foothills. In the intermediate region, where the Beskid range intersects with the south-north axis of elevated potassium radioactivity, the values are well described by the average of the megaregion.

**Fig. 4:**
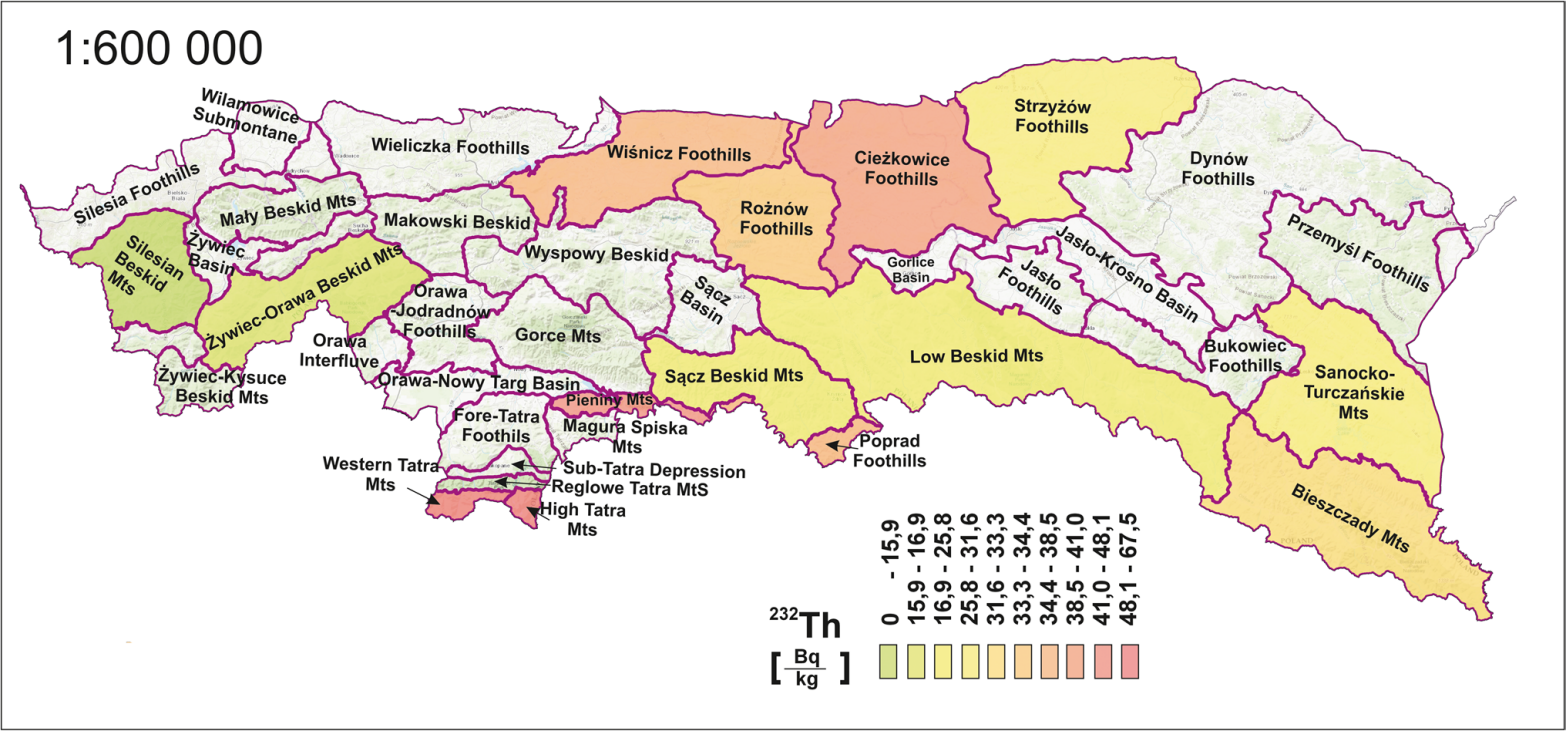
Spatial distribution of ^232^Th radioisotope concentrations by mesoregion

Figure 4 presents the distribution of radioactivity in the soil for radioisotope ^232^Th. The average value calculated for the Polish Carpathian was 36 Bq·kg^−1^, and the values for individual mesoregions were in the range of 14–67 Bq·kg^−1^. In other parts of the Carpathians located in adjacent countries, the radioactivity of ^232^Th in soils is slightly higher compared to the results obtained. In Slovakia, ^232^Th ranges from 1.22 to 117 Bq·kg^−1^, with an average of 42 Bq∙kg^−1^ (Porubčanová et al. 2015) while in Romania for ^228^Ac (a secular equilibrium with ^232^Th can be assumed) 19–85 Bq·kg^−1^ with an average of 46.7 Bq·kg^−1^ (Blebea-Apostu et al. 2024).

The distribution within the area of research was similar to that described for the ^40^ K radioisotope, in proportion to the mean concentrations. However, the foothills in the north (Ciężkowice, Wiśnicz, Rożnów) are the most closely aligned around the average in this comparison. This is directly related to the high radioactivity of the thorium series in the Tatras Mountains, where the average for all other regions is more than twice as low.

**Fig. 5:**
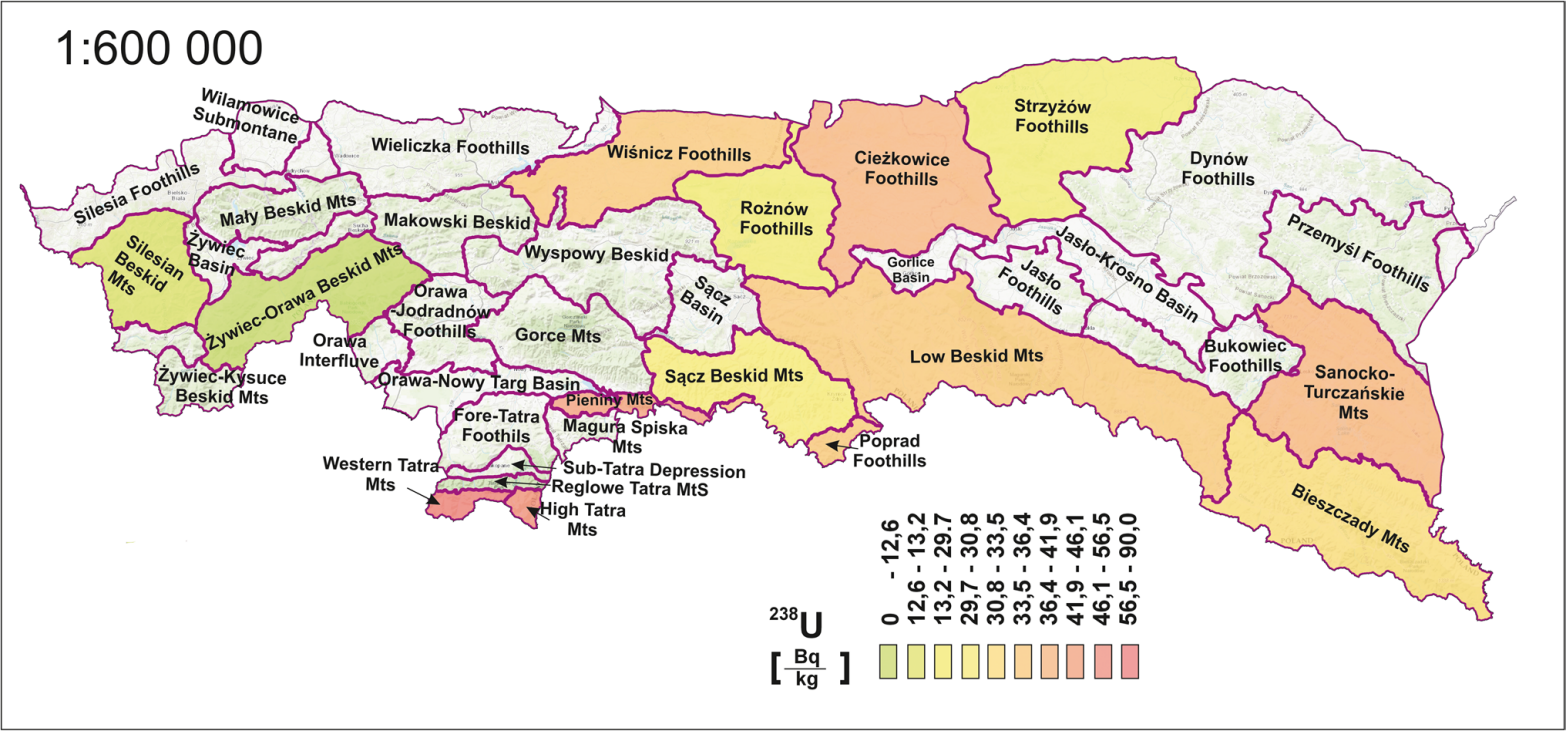
Spatial distribution of ^238^U radioisotope concentrations by mesoregion

Figure 5 presents the distribution of radioactivity in the soil for the ^238^U radioisotope. The distribution was similar to that of the ^232^Th. Again, radioactivity in the Tatras was at much higher levels. The average value of the radioactivity concentration in the soil of the Tatra mesoregions alone was 73 Bq·kg^−1^, while for the other mesoregions, it was 32 Bq·kg^−1^. The total radioactivity concentration of ^238^U was in the range of 13 to 90 Bq·kg^−1^. The results of ^238^U radioactivity concentration were compared to other Carpathian regions. The content of ^238^U in the Slovak Carpathians is in the range of 10–146, with an average of 51 Bq·kg^−1^, while in the Romanian Carpathians ranges from 15 to 126, with an average of 54 Bq·kg^−1^ (Blebea-Apostu et al. 2024; Porubčanová et al. 2015). These data also show that in the Polish Carpathians, the concentration of ^238^U is slightly lower than in Slovakia and Romania.

The Tatra mesoregion again had the highest radioactivity, but when comparing the distribution of ^232^Th and ^238^U concentrations independently for all other mesoregions, some insignificant differences can be seen. For this part of the study area, it is again possible to identify the same regions that deviate significantly from the average value. Values are significantly lower in parts of the western Beskidy (Silesian, Zywiec-Orawa), and highest in the southern (Pieniny). In other mesoregions, the deviation from the mean value does not exceed one standard deviation.

Based on the radioactivity concentrations of radioisotopes ^40^ K, ^232^Th, and ^238^U, the absorbed dose rate (ADR) and the annual effective dose (AED) were calculated. These are convenient by reducing isotope concentrations to a single parameter that can be used to interpret the overall distribution of radioisotopes in the study area. The average ADR and AED values were 58.8 nGy·h^−1^ (23.6–104.9 nGy·h^−1^) and 0.11 mSv·y^−1^ (0.04–0.19 mSv·y^−1^), respectively, for the Polish Carpathian megaregion. The AED parameter is recalculated directly from the ADR based on the assumption of average time spent outdoors, which was made according to UNSCEAR recommendations (UNSCEAR 2008). Therefore, the values of both quantities are proportional and can be collectively analysed on either value.

The result can be related to global averages, where the dose distribution for the megaregion is consistent with that worldwide mean value and elevated relative to the average reported for Poland. For Poland and the world, the average ADR is 45 nGy·h^−1^ and 59 nGy·h^−1^, respectively. The observation from the data also indicated relatively wide variations occurring between the extreme values. However, in relation to global ranges, only 10% of the population lives in areas with an absorbed dose rate of terrestrial gamma radiation below 26 nGy·h^−1^ and 7% above 92 nGy·h^−1^ (UNSCEAR 2008). The results for Polish Carpathians are also within the Europe range (Chandra et al. 2023).

**Fig. 6:**
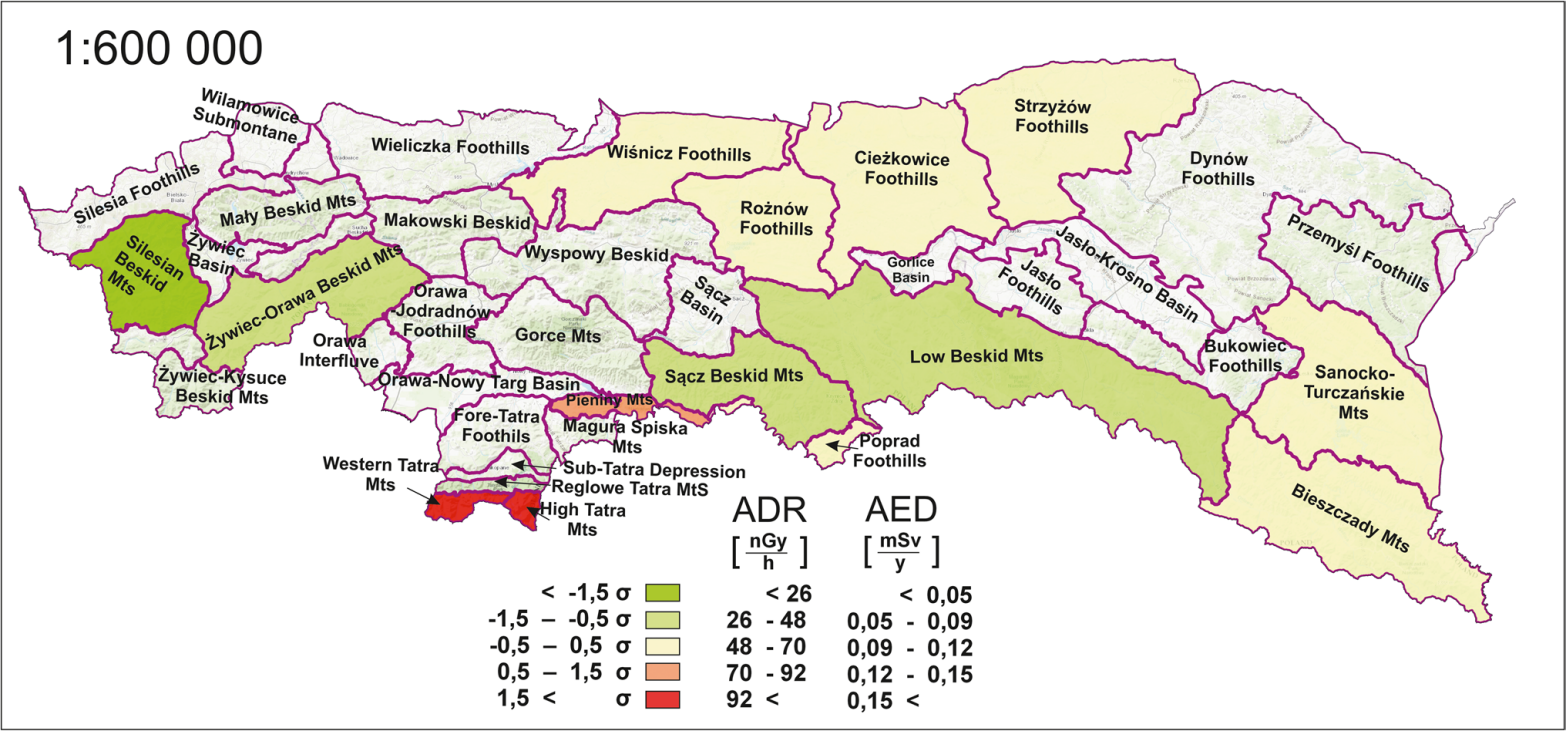
Spatial distribution of absorbed dose rate (ADR) and annual effective dose (AED) by mesoregion

Figure 6 presents the distribution of the ADR and AED parameters, including the symbolisation of the polygons based on the standard deviation. This allowed the identification of areas with lower and higher exposure of residents. Analysing the map presented, it is possible to distinguish three areas that are quite consistent in their location. The first area is the Beskid range, through the central part of the megaregion, which presented statistically below-average values. The second area is in the southern part, where the values were above average. The dose distribution in the northern and eastern parts of the Carpathian range was approximately comparable to the average value, where the third region could be recognised. The maxima and minima were in the Tatra and Silesian Beskid mesoregions, respectively.

The following part of the study focused on analysing the various factors that could influence the presented dose distribution. Therefore, the ADR map was related to the available GIS data, e.g. soil type and geology.

### Correlation between geological aspects and absorbed dose rate distribution

**Fig. 7:**
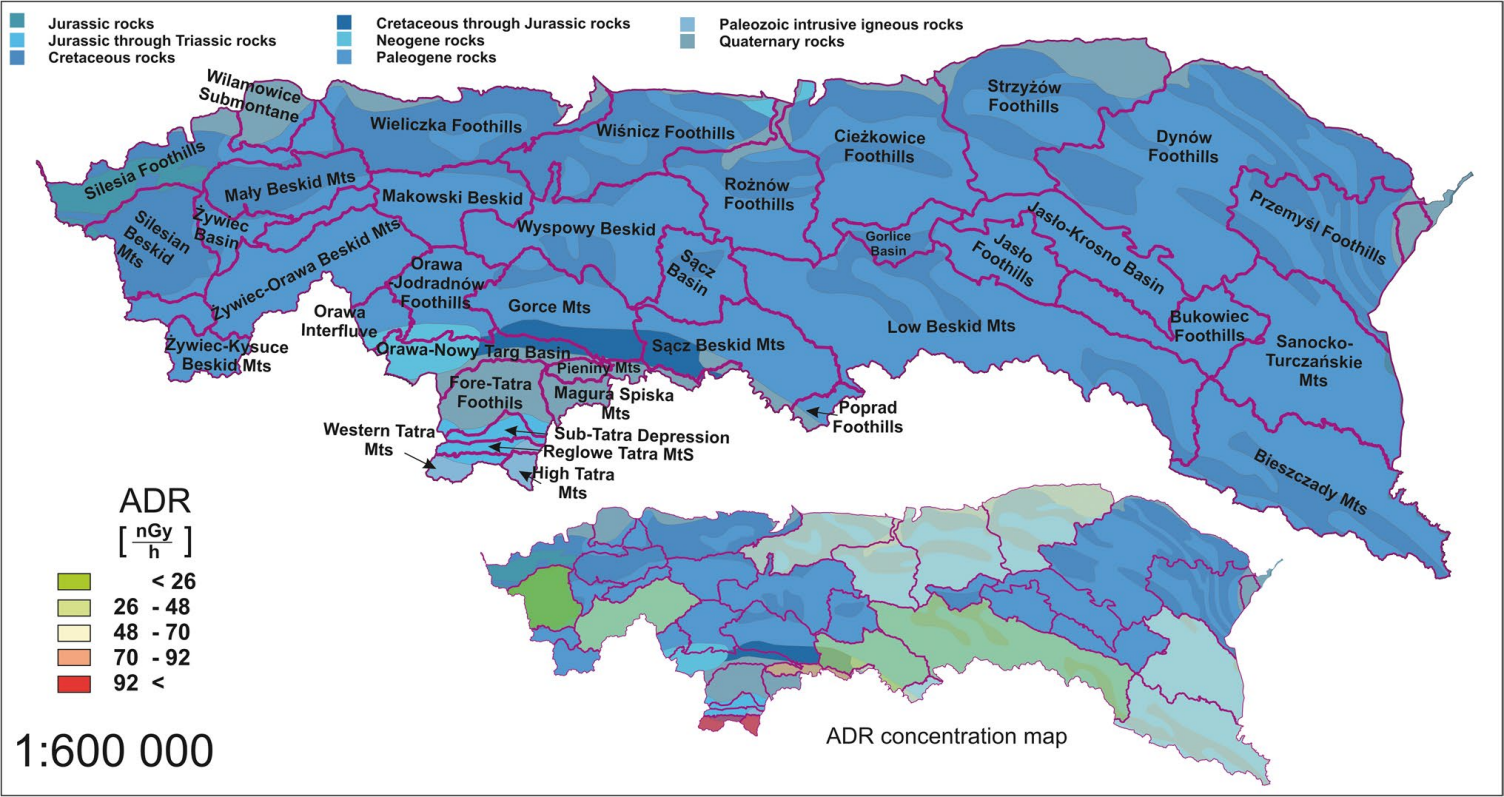
The generalised geological age of surface outcrops of bedrock compared to the ADR distribution

An important aspect determining the radioactive concentration of terrestrial radioisotopes in the soil is the bedrock. The ADR distribution map was compared with the geological age of the surface outcrops of bedrock layer in Fig. 7 (Pawlewicz et al. 2003). The correlation between the geological age of the bedrock and the radiological characteristics was checked. The vast majority of the mesoregion’s bedrock consists of Paleogene-dated rocks. A general relationship was observed; the ADR value is inversely proportional to the percentage of peleogenic rocks. An exception to this rule was the Silesian Beskid mesoregion, where the Paleogene-dated bedrock cover was only 9%, and ADR was the lowest of all mesoregions at 23.6 nGy·h^−1^. The second case was the mesoregion of the Sanocko-Turczańskie mountains, with 97% of the Palaeogene formations; the ADR was 56.0 nGy·h^−1^. For the other points, the relationship was almost linear, where the percentage contribution of rocks of a different geological age is reflected in the increased dose rate. The distinct effect on the increase in dose was related to Cretaceous rocks, Quaternary rocks, and Palaeozoic intrusive igneous rocks. For the area of the Tatra Mountains with the highest ADR, the contributions of Palaeozoic rocks were 82% and 99%, respectively, for the mesoregions of the Western Tatra and the High Tatra. Palaeozoic-dated rocks were not present in other regions, for which an increased proportion of Quaternary or Cretaceous rocks influenced the dose rate power. In general, from the perspective of the geological age of the bedrock, certain factors have been identified that may be relevant parameters according to ADR. It is possible to distinguish some similarities within the three areas of ADR variation highlighted earlier. In particular, it can be emphasised that the rocks formed in the Palaeogene are characterised by a low level of radioactivity. Nevertheless, some mesoregions were also observed, where the measured ADR value could not be explained on the basis of the above correlations, e.g. the Silesian Beskid.

For areas of similar orogenesis, the age characteristics of the bedrock appear to be a relevant factor for radiological characterisation. Different geological ages are associated with characteristic geological processes, and often, it could be related to distinct types of rocks. However, it provides only indirect information and is rather a secondary parameter to the more precise rock formation characteristic.

**Fig. 8:**
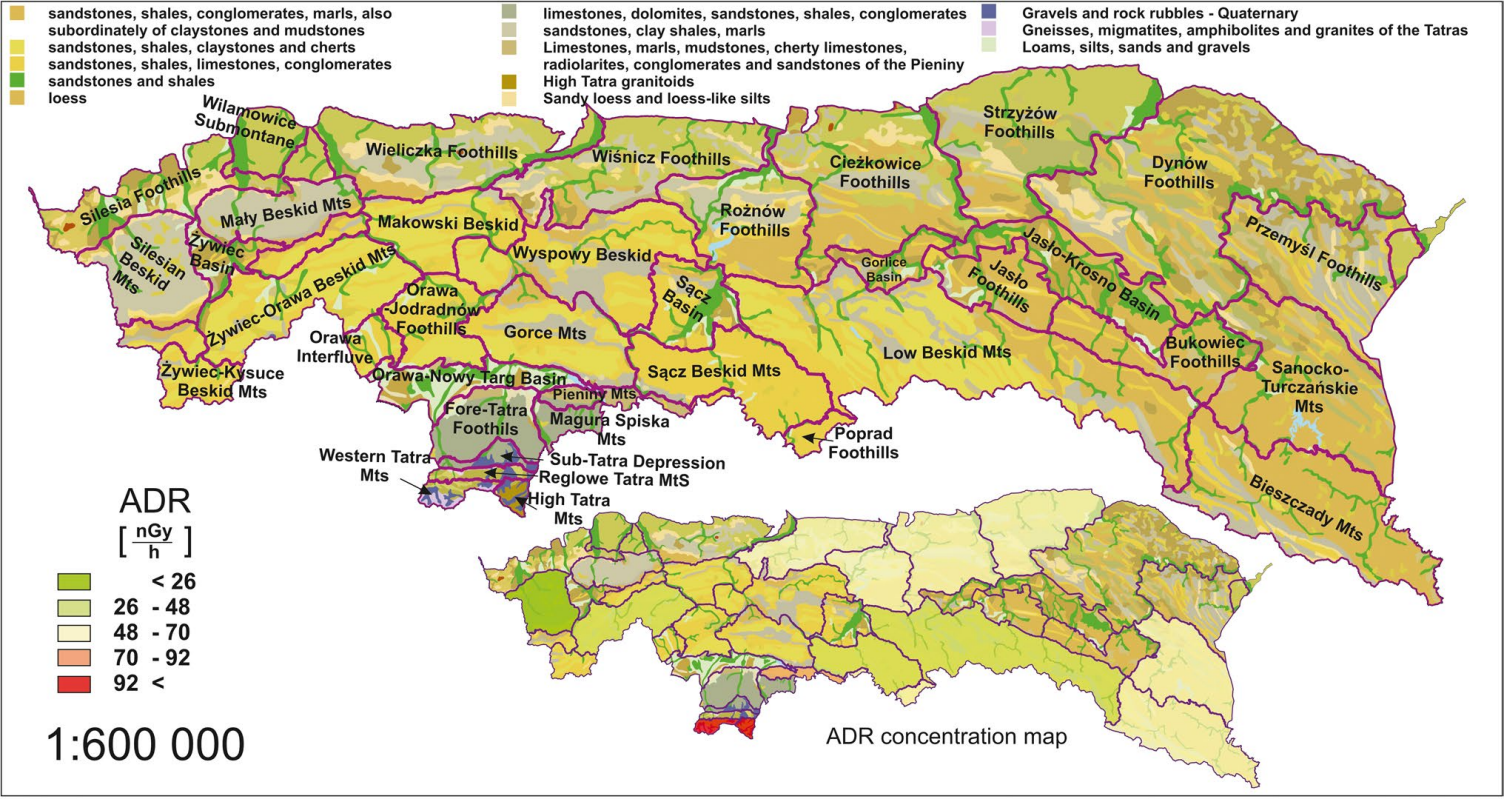
The surface geological structure compared to the ADR distribution

Figure 8 shows a more detailed map of the rock formations that compose the surface bedrock (PGI PGINRI 2022). Due to the fact that the authors only provided partial data, it was not possible to indicate the correlation with direct percentages of a particular rock type for the individual mesoregions. However, using visual interpretation, it is possible to identify important components that may influence the radioisotope content of soil formed on a particular bedrock.

Geological units that occur in significant contributions in the Tatras mesoregions of the highest ADR were granitoids and granites, gneisses, migmatites, amphibolites, quaternary gravels, and rock rubbles. The occurrence of these rock formations was limited to the Tatra region and was the possible reason for the significant dose increase. Another point with an elevated ADR parameter was in the Pieniny Mountains, where the lithography of the area consists mainly of limestones, marls, mudstones, cherty limestones, radiolarites, and conglomerates. It should be noted that the rocks, according to the authors of the map presented in Fig. 8, should be dated to the geological period of Cretaceous through Jurassic rocks. This is in contrast to the dating presented in Fig. 6, where 99% of the mesoregion site was represented by Quaternary rocks, but there, the studies were carried out at a lower resolution.

In contrast, the mesoregions with the lowest ADR were characterised by a high fraction of sandstones, shales, limestones, claystones, clay shales, and conglomerates. The point with the lowest absorbed dose rate consisted mainly of sandstone and shale.

The intermediate ADR value was observed for regions where, in addition to sandstones and shales, the contributions of loess, marls, and clay minerals were observed.

Again, three regions with relatively consistent characteristics can be visually distinguished, which corresponds to the distribution of ADR in the study area.

In general, the lithographic characteristics of the mesoregions are very complex, and it is difficult to refer to specific mineral fractions within the geological divisions. However, it is possible to see a clear increase in the dose rate for igneous rocks and lower values for sandstones. Blebea-Apostu et al. presented results from similar work for the Romanian part of the Carpathian, and the findings are comparable. The soils formed in granites and granitoids were characterised by one of the biggest ADR, when sandstones were the lowest (Blebea-Apostu et al. 2024). This view is supported also by authors in works from areas different from the Carpathian region, for instance, R. O. Baston and C. R. Appoloni. In their article, the ADR for igneous rocks averaged 148 nGy·h^−1^ while for sandstones 17 nGy·h^−1^ (Bastos and Appoloni 2009). These two rock types are given as mostly having extremes of absorbed dose rate or overall radioactivity, and confirmation of this relation can be found in numerous literature positions (Bastos and Appoloni 2009; Bastos et al. 2008; Marshall 1999; REIMANN 2000a; Sanjurjo-Sánchez and Alves 2017; Schmus 1984). However, there are no simple rules when it comes to different rock formations. Soils formed on limestone, for example, are characterised by a highly individual absorbed dose rate. In the Pieniny mesoregion, where the bedrock consists almost entirely of limestone and sedimentary rock formations, the ADR rate was relatively high, averaging 77 nGy·h^−1^. For the Romanian part of the Carpathians, the value was similar at 79 nGy·h^−1^ (Blebea-Apostu et al. 2024). Nevertheless, reviewing the literature from global studies (including South America, Africa, Asia, and Europe), it ranges between 23 and 132 nGy·h^−1^ (Ahmad et al. 2020; Ahmed 2005; Brígido Flores et al. 2008; Chowdhury et al. 1998; Sharaf et al. 1999; Trevisi et al. 2012; Turhan 2008; Xinwei 2005).

### Correlation between soil type composition and absorbed dose rate distribution

**Fig. 9:**
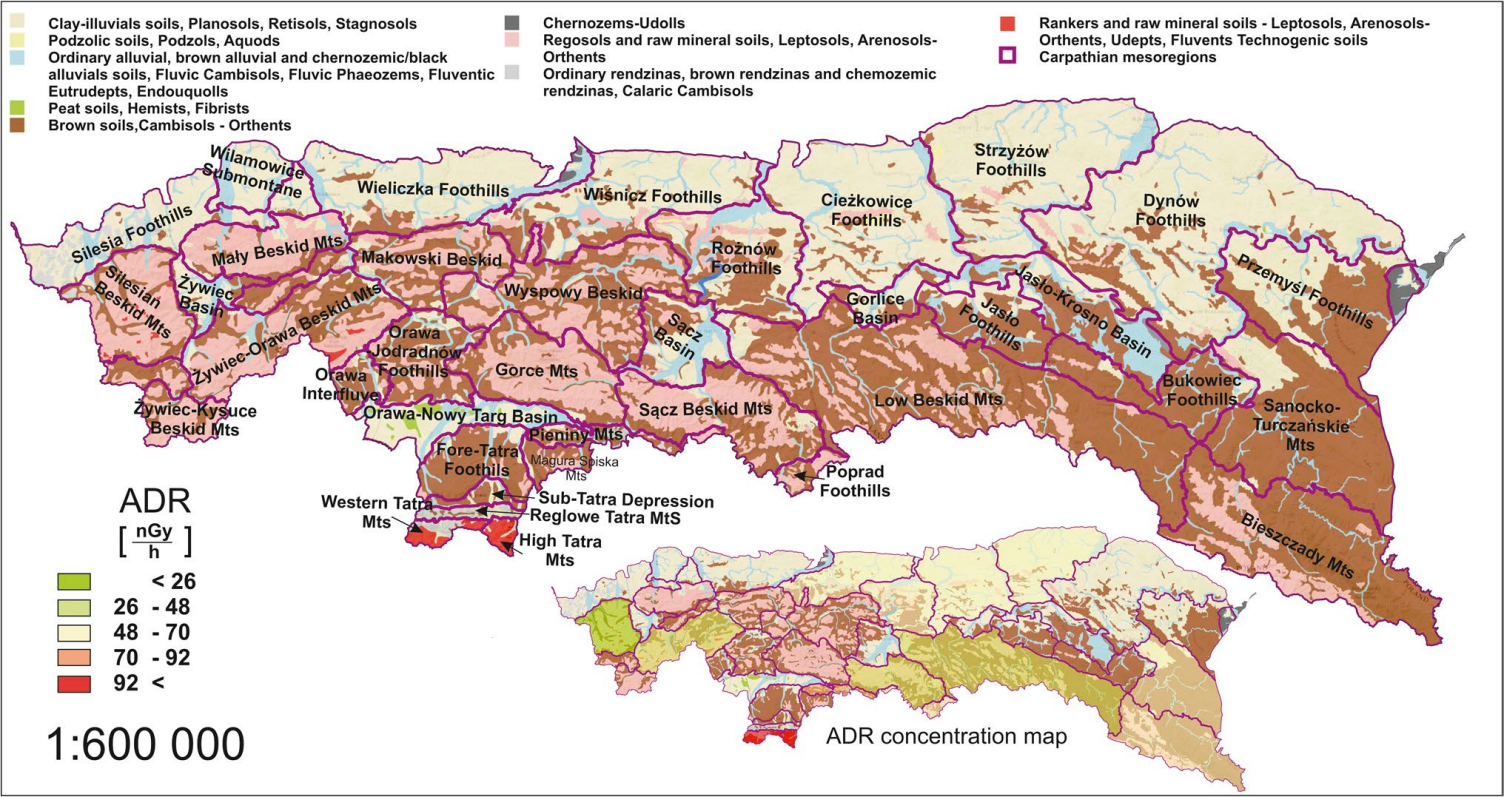
Spatial distribution of soil types compared to the ADR distribution

A correlation was made between the main soil types that cover the mesoregion area and the absorbed dose rate. Figure 9 presents a map of the distribution of individual soil units in the study area based on mapping on a scale of 1:300,000 (Dobrzański et al. 1984). A large part of the area has soils in the nature of raw mineral soils and poorly developed soils, characteristic of mountainous areas. Particularly in the southern part, the soils are predominantly leptosols, that is, poorly developed soils, shallow, on rocky ground, characterised by a low organic matter content. The map shows the predominant location of this soil formation within the area with the highest ADR, the Tatra mesoregions. However, in contrast, the areas with the lowest ADR are mainly covered by soils with similar characteristics, also leptosols and less developed raw minerals—regosols. These are generally low in organic matter, sandy soils. Soils with these characteristics indicate the intensity of erosion processes. Along the central range of the Beskidy, the proportion of brown soils is increasing. These are more developed soils, characterised by the presence of a brown humus layer on the surface, which contains large amounts of organic matter. The largest coverage of brown soils occurs in the mesoregion of the Sanocko-Turczańskie Mountains. On the northern side, for the entire Carpathian megaregion, a transition to clay-illuvial soils is observed. Soils of this type are characteristic of regions with high hydrological activity, where rainwater leaches carbonates and clay minerals. The presence of clay in lower horizons can contribute to improved soil structure, providing better water retention and nutrient availability compared to soils with uniform clay distribution. From the point of view of the radioisotope content of soils, it is difficult to find any correlation with soil type. A high soil content with the weakest formation level (high content of skeletal fractions) can be observed for both the lowest and highest ADR areas. However, the type of soil indicates mostly the intensity of the erosion process. NORM content in bedrock is more of a crucial indicator here. A stronger correlation to the composition of the underlying rock is more predicted.

Taking into account the worldwide literature, it is hard to make a comparison via soil type, because of the different structures of the area and the different groups of the soil used. Mineral soil can be stated to be characterised by higher radioactivity compared to others (Luiz do Carmo Leal et al. 2020). Podzols are characterised by the lower radioactivity of natural radioisotopes, which can combine with a low ADR level. Cambisols as leptosols are a component of a few types of soils. Based on their radioactivity, it can be estimated that the ADR will be at a comparable level (Ribeiro et al. 2018).

Previous subsections have used the ADR coefficient to look for correlations between different environmental factors. It was also decided to check to what extent the presented correlations are reflected in the individual components of the ADR parameter, i.e. radioisotopes of the uranium series, thorium series, and ^40^ K. It was checked how much the individual components contribute to the ADR factor, considering the radioactive concentration of the isotopes in the samples. On average, for all 110 measurement points, the shares were as follows: 34 ± 9% for ^40^ K; 30 ± 10% for the uranium series; 37 ± 5% for the thorium series. Therefore, if the proportions are relatively equally distributed, then the ADR relationships presented should be universal for the rest of the isotopes.

### Mesoregionalisation compared to macroregionalisation

**Fig. 10:**
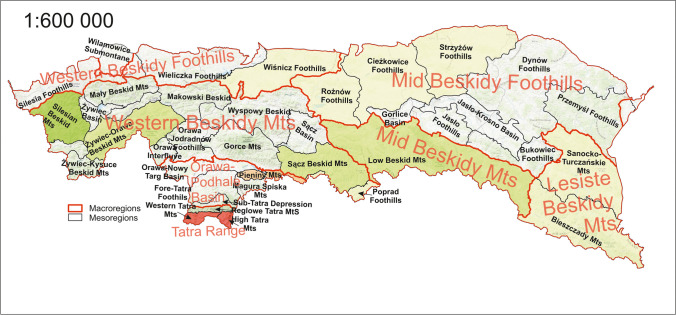
Spatial distribution of absorbed dose rate (ADR) in mesoregions and macroregions

The distribution of the ADR ratio shows that some interrelationships can be observed that extend beyond the boundaries of the mesoregions. Analysing previous comparisons, it may be concluded, that similarities are best reflected in lithographic and geochronological mapping of the area. The analysis against the soil data in this case did not result in any significant visible remarks. Relating the above observations to the physiographic study of the Polish Carpathian site and its regionalisation, Fig. 10 shows the distribution of ADRs with the macroregional units also included. The macroregional divisions in the referenced study were established based on the following characteristics: geographical location, nature and origin of relief, and lithological diversity. At the level of mesoregional division, the key factor was morphogenetic, which is mainly based on the consistency of exogenic processes shaping landform relief. Consequently, the differentiation between mesoregion and macroregion is mainly due to the intensity of climatic factors including weathering, denudation, erosion, and sedimentation. Therefore, consistency in the distribution of ADR is visible, between mesoregions, within a single macroregion. For example, the difference between the ADR values for the macroregion ‘Lesiste Beskidy Mountains’ is less than 1 nGy·h^−1^. The greatest variability within the macroregion was observed for the ‘Western Beskidy Mts’, where the measure of variation, quantified by the standard deviation for a set of mesoregions, was 10 nGy·h^−1^. Given the large area of 5500 km^2^, compliance is very good.

## Conclusions

Based on the results, it can be concluded that:

1. In the study area of the Polish Carpathians, there were no significant anomalies in the absorbed dose rate of terrestrial origin. The results for each subdivision were within the ranges found worldwide, where a number of reports and publications were used for comparison. The average for the study region was at the same level as the global average.
2. The study area turned out to be very diverse. The observed ADR values occurred within both the lower and upper maxima of the global distribution. This makes the area very interesting to analyse the distribution of individual radioisotopes and the absorbed dose rate.
3. Physiographic mapping was used in the distribution analysis. It was based on the division into mesoregions, when it was eventually found that mesoregions within a macroregion showed similar correlations. In the division into macroregions, the key aspect is consistency in terms of the nature and origin of the relief and lithological diversity. In this regard, consistency can also be expected in terms of radiological characteristics. In particular, when the main component of the dose is radioisotopes of rock origin.

In summary, the work shows a new strategy that can be adapted to determine sampling points when monitoring human exposure to ionising radiation. Division into larger units with consistent characteristics allows to reduce the number of points of collection of material for testing. Therefore, based on the results, it is possible to consider that physiographic maps and, in particular, the macroregional division, could be very helpful. By using such divisions, the workload in this kind of research is also reduced. Finally, by reducing sample points, the time to obtain results is also reduced, and the exposure levels can be calculated in a shorter time.

## Data Availability

The data that support the findings of this study are available from the corresponding author, Filip Jędrzejek, upon reasonable request.
